# Use of Infrared Thermal Imaging for Assessing Acute Inflammatory Changes: A Case Series

**DOI:** 10.7759/cureus.28980

**Published:** 2022-09-09

**Authors:** Jose L Ramirez-GarciaLuna, Karla Rangel-Berridi, Robert Bartlett, Robert DJ Fraser, Mario A Martinez-Jimenez

**Affiliations:** 1 Surgery, Hospital Central Dr. Ignacio Morones Prieto, San Luis Potosí, MEX; 2 Experimental Surgery, McGill University, Montreal, CAN; 3 Emergency Medicine, Swift Medical, Toronto, CAN; 4 Medical Education and Simulation, Swift Medical, Toronto, CAN

**Keywords:** allergy, trauma, vasodilation, inflammation, thermography, infrared thermal imaging

## Abstract

Infrared thermal imaging is a non-contact imaging modality that captures the heat emitted by the human body. Thermal regulation or heat load to the different body parts is mainly regulated via blood supply, which is increased during inflammation. The assessment of the body's level of inflammation with pain, erythema and heat is subjective clinical measurement. Infrared imaging can be an objective tool for identifying and following inflammatory and perfusion changes, thereby helping clinicians locate and document the extent of the inflammation as well as monitor the response to treatment. As an example of this, here, we present three clinical cases where the use of thermography aided the assessment of acute inflammatory changes due to trauma, vasodilation, and allergy.

## Introduction

Infrared thermal imaging (IRT) is a non-contact imaging modality that captures the heat emitted by the human body. This technology is based on the detection of heat as part of the infrared electromagnetic spectrum and has been demonstrated to be helpful in the assessment of different medical conditions, particularly those associated with inflammatory or perfusion changes [1,2]. IRT is well-suited for assessing these changes because the human skin acts as a black body, meaning that approximately 98% of the infrared radiation it emits represents its thermal load and less than 2% consists of external radiation reflected by the skin [2,3]. Thus, IRT can provide an objective measure of the skin's temperature and underlying tissues and their physiological blood flow regulation under normal and pathological conditions [4,5].

The release of cellular mediators following tissular insults leads to the cardinal signs and symptoms of inflammation described by Celsus: rubor (erythema), dolor (pain), tumor (increase in volume due to edema), and calor (increased heat load) [6]. However, because these signs and symptoms are subjective, their clinical assessment is prone to bias and errors due to the clinician's experience and the patient's perception. Subjective measurements lead to poor accuracy and repeatability; thus, objective measurements such as those provided by IRT can help in the assessment of injuries and to make more informed clinical decisions. Here, we present three clinical cases where IRT images were acquired over time in patients suffering from trauma and allergy testing. These cases illustrate how IRT adequately images the thermal load created by vascular changes and how it can be used to monitor and assess different patient conditions. In all cases, the images were acquired using a Ray 1 hyperspectral imaging device (Swift Medical, Toronto, ON) after undressing the affected areas for a minimum of five minutes to allow reaching thermal equilibrium, at a distance of 30 cm, under controlled lighting, temperature and humidity conditions, and following the Thermographic Imaging in Sports and Exercise Medicine (TISEM) checklist for IRT imaging [7]. The resulting IRT image set consists of a thermogram showing the absolute temperature of the body regions and one where a control region of healthy tissue is selected, and the images are scaled to this region of interest (relative temperature). The use of relative temperature for the assessment of inflammatory and infectious processes has been demonstrated to be superior to the use of absolute temperature thermograms [8]. Thus, we refer the reader to this set of images for this study. This study was conducted according to the requirements of the Declaration of Helsinki (64th World Medical Association General Assembly, Fortaleza, Brazil, October 2013), and all patients provided informed consent for preparing and publishing this case report.

## Case presentation

Case 1: Ankle sprain

**Figure 1:**
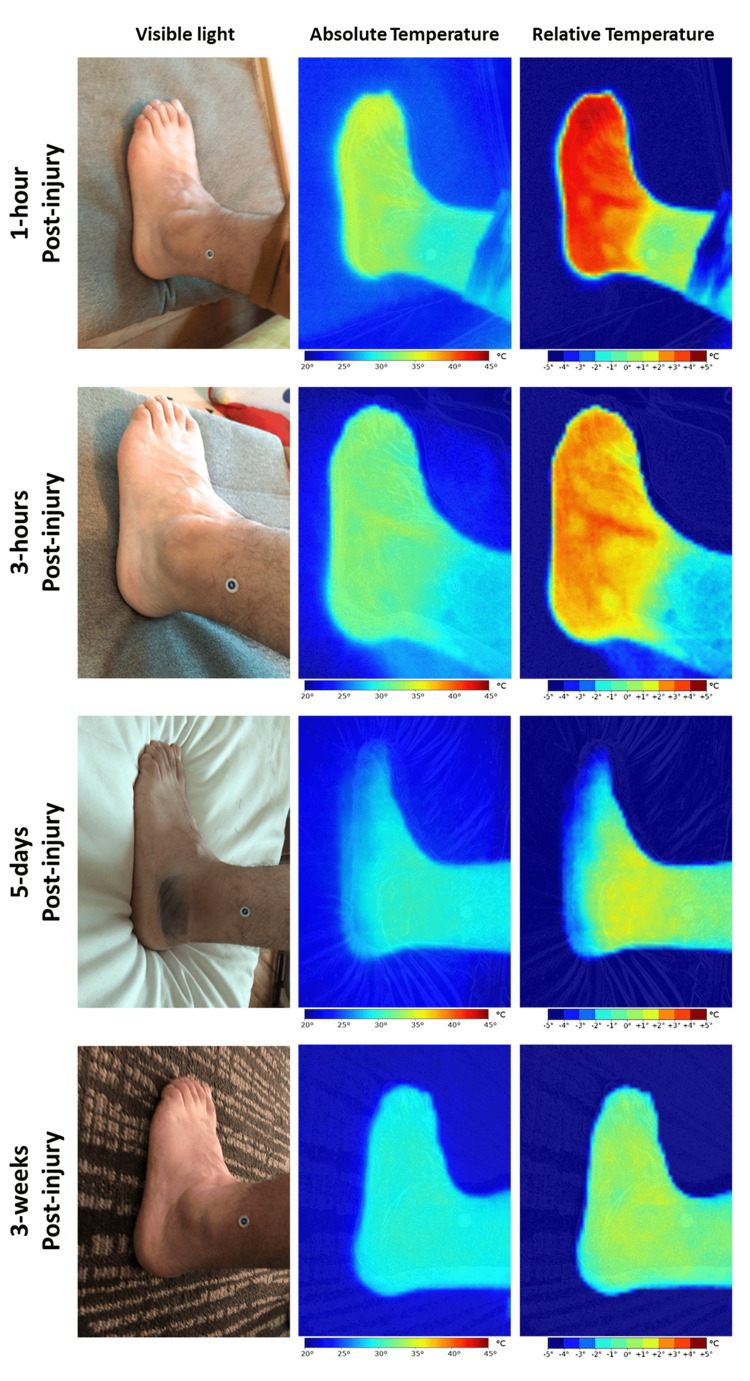
Ankle sprain Clinical images and thermograms of the affected foot were acquired at one and three hours post-injury in the emergency department and five days and three weeks later at the office. Acute inflammatory changes were easily observable during the day of the injury, as well as vascular trajects within that area. Residual inflammation was cleared on day 5 post-injury and completely disappeared by three weeks. Images were taken with a Ray 1 hyperspectral imaging device (Swift Medical, Toronto, ON).

An otherwise healthy 36-year-old male presented to the emergency room (ER) with intense pain in the left ankle following a sprain injury. On presentation, the lateral aspect of the ankle had localized swelling over the malleolus and there was increased foot temperature under palpation. The patient referred severe pain and paresthesia of the foot. Otherwise, no obvious deformity or asymmetry was noted during the clinical examination. While the active range of motion of the foot was decreased due to pain, the passive range of motion was considered normal, and the patient was able to tolerate weight bearing as he had come walking into the ER. The neurovascular examination was normal, and strong pulses were detected. Ankle external rotation, syndesmotic ligament palpation, and proximal tibiofibular squeeze tests ruled out a syndesmotic fracture. Thus, following the Ottawa Ankle Rules, no further radiological testing was requested, and a grade II lateral ankle sprain was diagnosed [9]. IRT images showed an increased thermal load of approximately +5˚C of the foot compared to the proximal aspect of the ankle (Figure 1). The patient was treated with non-steroidal anti-inflammatories and an ice pack. After two hours, the thermal load was significantly reduced, and the patient referred less pain; therefore, he was discharged, and immobilization with a controlled ankle movement (CAM)-walker boot was prescribed, along with rest, ice, and anti-inflammatories. IRT imaging at follow-up (five days and three weeks after the injury) showed no more thermal anomalies on the foot, highlighting how the initial "hotspots" were induced by vasodilation caused by the injury.

IRT imaging for assessing ankle sprains has been used in the past, showing a modest correlation between the temperature and the patient-referred symptoms, namely pain [10]. However, more research needs to be conducted into whether this imaging modality can offer a correlation to severity scores or to other diagnostic imaging methods such as x-rays.

Case 2: Allergy testing

A 31-year-old male with a history of asthma and atopic dermatitis was sent for skin prickle allergen testing as part of his routine assessment. Skin prickle allergy testing consists of the evaluation of type 1 immunoglobulin E (IgE)-mediated immune response to allergens. Type 1 allergic reactions frequently present with cutaneous, nasal, respiratory, ocular and sometimes gastrointestinal symptoms that include wheezing, shortness of breath, nasal congestion, swelling and tearing of the eyes, swelling of the lips, itching, hives, abdominal discomfort or diarrhea caused by vasodilation and angioedema secondary to mast cell and basophile activation. During the skin prickle test, diluted allergens are introduced using a sterile needle into the skin at least 2 cm apart on the flexor aspect of the forearm along with a positive (histamine) and a negative (saline) control. The test area is examined after 20 to 30 minutes to evaluate the skin reaction. The presence of a wheal with a diameter of 3 mm or greater is considered indicative of a positive reaction, with larger wheals indicating higher sensitivity to the allergen. Finally, an oral antihistamine is administered at the end of the test to revert the signs and symptoms created [11].

**Figure 2:**
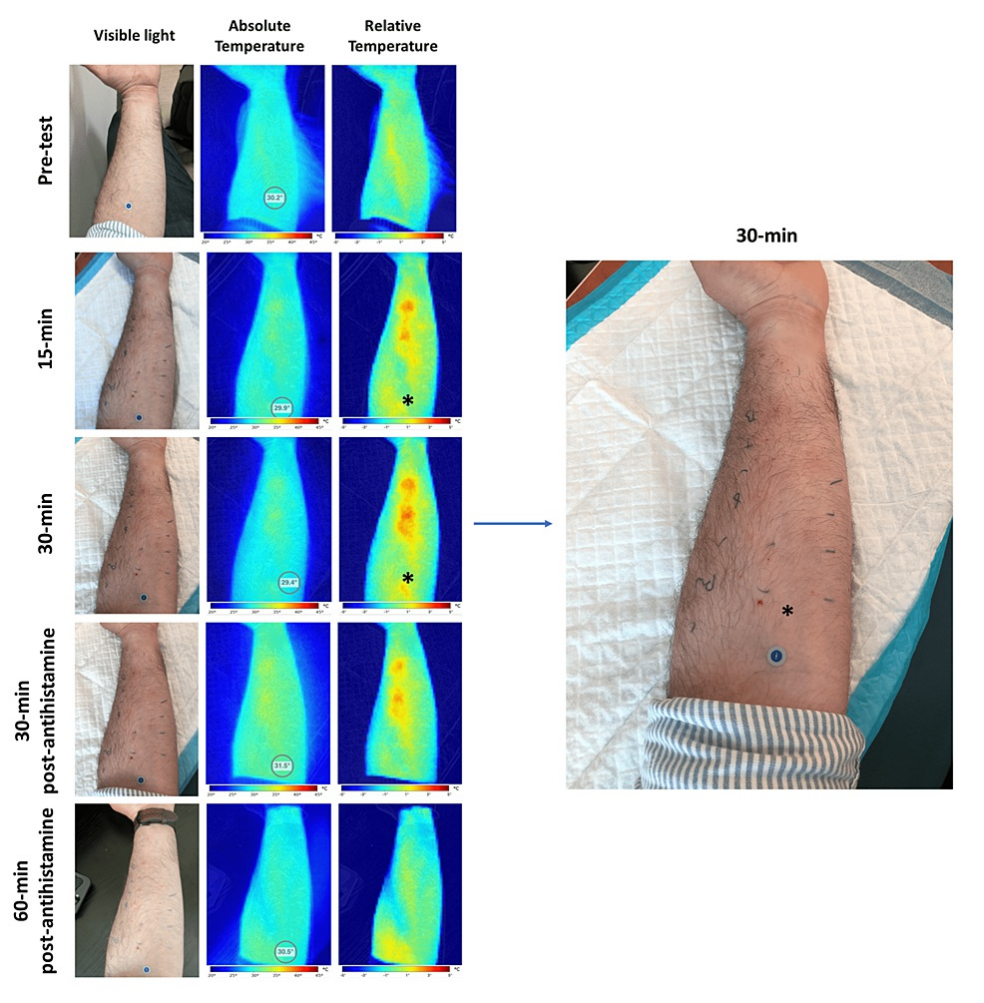
Allergy testing An allergy scratch test was performed on a patient with a history of asthma and atopic dermatitis. Pretest thermograms show an even temperature distribution. Vasodilation and inflammation were apparent after the antigen administration, with a peak inflammatory response at 30 minutes post-administration. Three zones presented more drastic changes than the positive control area of inoculation (asterisk), which were easily identifiable under thermal imaging. However, the visible light image (insert panel) only showed moderate erythema in those regions. All inflammatory changes were reversed after the administration of an antihistamine. Images were taken with a Ray 1 hyperspectral imaging device (Swift Medical, Toronto, ON).

In the case of our patient, IRT images were acquired before administering the allergens and at 15 and 30 minutes post-inoculation (Figure 2, upper panels). The images show the areas of maximal inflammatory changes, which correlate with wheal and erythema on the visible light images and are even more intense than the signal produced by the histamine control (asterisks). Interestingly, they also allow the quantification of the inflammatory response both as a function of the local thermal load and the area of the ensuing "hotspots." The lower panels of Figure 2 show how the inflammatory changes reverted after antihistamine administration. The use of IRT for assessing allergy skin prick tests demonstrated a sensitivity of 72%-93% and a specificity of 60%-88%; thus, this imaging modality can be used to automatize and objectivize the test procedure particularly when paired with computer visioning AI techniques [12,13].

Case 3: Trauma injury

**Figure 3:**
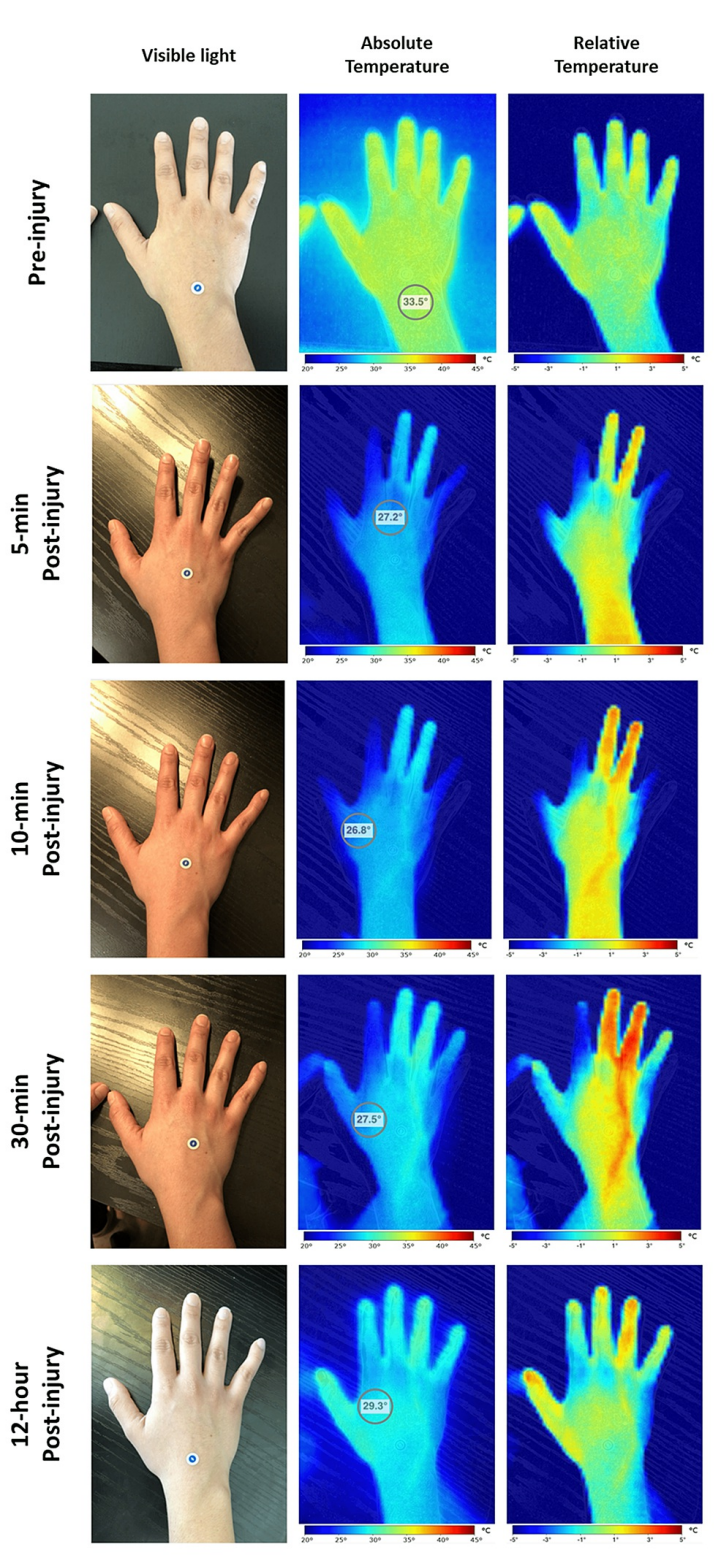
Trauma injury Pre-injury thermograms of a subject's hand show a homogeneous temperature distribution. Time-lapse images acquired after a minor crush injury to the finger show buildup of inflammation in the injured area and the clearance of them through the dorsal veins. In addition, blood flow redistribution from the other fingers became apparent. After 12 hours, most changes disappeared and the temperature distribution tended to a more homogeneous one. Images were taken with a Ray 1 hyperspectral imaging device (Swift Medical, Toronto, ON).

One of our research team members sustained a minor crush injury to her right hand's third and fourth fingers with a drawer. IRT images of her left hand before the injury obtained as controls show a uniform heat distribution on the fingers and volar aspect of the hand (Figure 3, upper panel). Time-lapsed IRT images after the injury showed the buildup of inflammatory mediators in the affected tissue and how they get removed by the venous blood flow (Figure 3, middle panels). In addition, the images showed a surprising redistribution of the blood flow in the non-affected fingers, resulting in "coldspots" on these areas. These phenomena highly suggest a broader physiological response to the injury and illustrate how IRT captures acute inflammation and dynamic vascular changes. The changes are also transitory, as the blood flow redistribution was reverted 12 hours after the injury, and the increased thermal load almost returned to its baseline (Figure 3, lower panel).

Previous studies have demonstrated a close correlation between blood flow in healthy tissue or wounds and their thermal load [14-16]. Increases in the blood flow are correlated with "hotspots," while conversely, reductions in it present as "coldspots" under IRT imaging [2]. Furthermore, differences in the temperature distribution, also known as thermal asymmetries, of bilateral body areas or areas that have multiple appendices such as the fingers in a hand, have been demonstrated to be helpful in the detection of injuries even before clinical signs and symptoms appear, as well as to be predictive of the severity of the lesions [17].

## Discussion

IRT is a point-of-care, low-cost, non-invasive, and non-contact imaging technology that offers powerful insights into the human body's physiology and response to injury. A growing body of evidence supports its use as an adjunct for detecting inflammatory or perfusion anomalies in diseases or conditions such as diabetes, cancer, infectious process, and trauma. IRT is well suited to provide objective measurements of inflammation that correlate well with clinically used scoring systems [18]. These objective measurements can then be used to monitor response to treatment, for example, when novel biological drugs or those with serious adverse events are needed. In addition, these objective measurements can also be used during clinical trials as effectiveness outcomes.

The commercialization of IRT technology has made it readily available as pocket-size devices to be used as stand-alone or integrated with imaging platforms enabled by smartphone technologies. In the latter case, the newer generation devices, such as the one used in our study, open the possibility of integrating IRT imaging with other advanced imaging modalities such as spectroscopy and bacterial fluorescence detection to enhance further its diagnostic capabilities for infection or perfusion anomaly detection [19,20]. An easy access to images allows serial imaging, which may enable better insights into pathological processes as well as treatment intervention efficacy. Thus, the combination of these technologies holds great promise for diagnosing and assessing the treatment response for wound care, and trauma and surgical patients.

Patient perspective

Patient 1 referred that seeing his foot “as an immense red blob” validated the amount of pain he was experiencing during his initial assessment at the ER. With previous injuries, the patient perceived that the healthcare team thought that he was exaggerating his pain. Previous research has demonstrated the effectivity of IRT as an adjunct to measuring pain since thermal variations correlate with autonomic responses [5].

Patient 2 was very interested in the inflammatory response shown in the IRT images. This patient had previously used IRT imaging for construction work to detect water leaks and faulty insulation but had never seen it be used for medical imaging. Both the patient and the nurse who applied and measured the response to the allergens agreed that IRT offered a more objective measure of inflammation than of the erythema and wheal response.

As a member of the research team, Patient 3 had seen IRT images of different pathologies. However, what she thought was the most striking in her images was the blood flow redistribution that occurred in the non-injured fingers. Despite the injury being minor, the vascular response was very intense. Furthermore, the time-lapsed images showed how the inflammatory response in the tip of the finger triggered, and the inflammatory mediators travel through the dorsal veins. Because these changes are transitory, the researcher felt fortunate to have been able to image the hand so soon after the injury.

In all three cases, the patients mentioned that IRT images offer easy-to-interpret images that help them understand what inflammation is, where it is being produced, and how it responds to treatment. They also mentioned that the images have the potential for patient education. In addition, Patient 1 mentioned that he felt the ER team did a better job explaining his injury and prognosis because of the images.

## Conclusions

In conclusion, IRT point-of-care imaging offers a deep insight into the physiology of tissue under normal and pathological conditions. Its use in the emergency department allows for a more thorough assessment of traumatic inflammatory injuries, monitoring response to treatment, as well as identifying other less obvious conditions. Its use requires relatively low-cost equipment and minimal training for image acquisition and interpretation. In addition to these benefits, it can also help with patient education and engagement with treatment, as highlighted in this report.
